# Choosing an appropriate somatic embryogenesis medium of carrot (*Daucus carota* L.) by data mining technology

**DOI:** 10.1186/s12896-024-00898-7

**Published:** 2024-09-27

**Authors:** Masoumeh Fallah Ziarani, Masoud Tohidfar, Mohsen Hesami

**Affiliations:** 1 Department of Cell & Molecular Biology, Faculty of Life Sciences & Biotechnology, Shahid Beheshti University, Tehran, 19839-69411 Iran; 2 Department of Plant Agriculture, University of Guelph, Guelph, ON Canada

**Keywords:** Artificial neural network, Genetic algorithm, In vitro culture, Machine learning, Multilayer perceptron, Radial basis function

## Abstract

**Introduction:**

Developing somatic embryogenesis is one of the main steps in successful in vitro propagation and gene transformation in the carrot. However, somatic embryogenesis is influenced by different intrinsic (genetics, genotype, and explant) and extrinsic (e.g., plant growth regulators (PGRs), medium composition, and gelling agent) factors which cause challenges in developing the somatic embryogenesis protocol. Therefore, optimizing somatic embryogenesis is a tedious, time-consuming, and costly process. Novel data mining approaches through a hybrid of artificial neural networks (ANNs) and optimization algorithms can facilitate modeling and optimizing in vitro culture processes and thereby reduce large experimental treatments and combinations. Carrot is a model plant in genetic engineering works and recombinant drugs, and therefore it is an important plant in research works. Also, in this research, for the first time, embryogenesis in carrot (Daucus carota L.) using Genetic algorithm (GA) and data mining technology has been reviewed and analyzed.

**Materials and methods:**

In the current study, data mining approach through multilayer perceptron (MLP) and radial basis function (RBF) as two well-known ANNs were employed to model and predict embryogenic callus production in carrot based on eight input variables including carrot cultivars, agar, magnesium sulfate (MgSO_4_), calcium dichloride (CaCl_2_), manganese (II) sulfate (MnSO_4_), 2,4-dichlorophenoxyacetic acid (2,4-D), 6-benzylaminopurine (BAP), and kinetin (KIN). To confirm the reliability and accuracy of the developed model, the result obtained from RBF-GA model were tested in the laboratory.

**Results:**

The results showed that RBF had better prediction efficiency than MLP. Then, the developed model was linked to a genetic algorithm (GA) to optimize the system. To confirm the reliability and accuracy of the developed model, the result of RBF-GA was experimentally tested in the lab as a validation experiment. The result showed that there was no significant difference between the predicted optimized result and the experimental result.

**Conclutions:**

Generally, the results of this study suggest that data mining through RBF-GA can be considered as a robust approach, besides experimental methods, to model and optimize in vitro culture systems. According to the RBF-GA result, the highest somatic embryogenesis rate (62.5%) can be obtained from Nantes improved cultivar cultured on medium containing 195.23 mg/l MgSO_4_, 330.07 mg/l CaCl_2_, 18.3 mg/l MnSO_4_, 0.46 mg/l 2,4- D, 0.03 mg/l BAP, and 0.88 mg/l KIN. These results were also confirmed in the laboratory

**Supplementary Information:**

The online version contains supplementary material available at10.1186/s12896-024-00898-7.

## Introduction

Carrot (*Daucus carota* L.) as a rich source of minerals, antioxidants, and dietary fiber is one of the most well-known root vegetables, which has been widely used for many biotechnological applications such as the production of recombinant proteins and functional genomics studies [1]. In vitro culture of carrot, in particular somatic embryogenesis, can be considered as a prerequisite for gene transformation and biotechnological studies [1]. However, developing and optimizing somatic embryogenesis protocols is a very costly and time-consuming process [2].

Somatic embryogenesis as a highly complex and non-linear process is influenced by different factors such as type and concentration of agar, medium composition (e.g., type and concentrations of salts and vitamins), type and concentration of plant growth regulators (PGRs), the pH of the medium, type and concentration of additives, type of vessel, the volume of the vessel, temperature, and light (quality and intensity) [3]. Therefore, it is difficult to predict and optimize somatic embryogenesis process by using conventional statistical methods such as simple stepwise regression [3]. Conventional statistical methods are typically employed for small datasets with limited dimensions [4–7]. One of the main demerit points of using conventional statistical methods is the high probability of overfitting. To tackle this impediment, machine learning algorithms can be used as an alternative mathematical method [8–11]. Different machine learning algorithms (e.g., artificial neural networks (ANNs), neuro-fuzzy logic systems, support vector machine (SVM), and random forest) have been recently used for modeling and predicting various in vitro culture systems such as explant sterilization [12, 13], in vitro seed germination [14], callogenesis [15–17], androgenesis [18], shoot proliferation [19, 20], rhizogenesis [21, 22], in vitro secondary metabolite production [8, 23, 24], and gene transformation [25, 26]. Among machine learning algorithms, different types of ANNs such as multilayer perceptron (MLP), radial basis function (RBF), and generalized regression neural network (GRNN) have been widely employed to model and predict in vitro culture processes [27].

MLP is a type of ANNs, which is applied for different aims such as clustering, predicting, and classifying the complex systems [12]. MLP is able to identify the relationship between target and input variables and recognize the inherent knowledge existent in the datasets without previous physical considerations [28]. However, MLP does not present a neat mathematical formula that illustrates the relative relationship of each independent variable in the model. Hence, MLP is considered as a “black box”. MLP consists of numerous highly interconnected processing neurons that work in parallel to find a solution for a particular problem [28]. MLP is learned by example. The examples should be carefully chosen otherwise time is wasted or even worse the model might be working inaccurately [28]. The main demerit point of MLP is that its operation can be unpredictable due to the fact that MLP learns how to find the solutions by itself [28]. On the other hand, RBF is a kind of interesting, powerful, and easy to interpret ANNs with supervised learning [19]. However, most plant tissue culture studies have employed the individual ANN, and the comparison between these ANNs has been rarely studied in the tissue culture area [27].

Genetic algorithm (GA) as the most well-known evolutionary optimization algorithm has been successfully applied to find the optimal level of factors involved in different tissue culture systems [27]. Indeed, a hybrid of machine learning and genetic algorithm can be considered as a robust mathematical method for modeling and optimizing different in vitro culture systems [27]. Therefore, ANN-GA can pave the way in modeling and optimizing carrot somatic embryogenesis.

The development of carrot somatic embryogenesis is a tedious task and needs the execution of complex experimental designs with numerous independent factors. This study evaluated whether ANN-GA can stably and accurately model and optimize carrot somatic embryogenesis. However, previous studies [29–35] selected the optimized level of factors involved in somatic embryogenesis by performing considerable bench work experiments. To overcome these challenges, in the current study, data mining by using ANN-GA was employed to model and optimize carrot somatic embryogenesis. We collected data from different carrot somatic embryogenesis studies. After modeling and optimizing the system, the result of ANN-GA was experimentally tested to confirm the reliability and accuracy of the developed model. To the best of our knowledge, this study is the first report of the application of data mining through ANN-GA in the field of carrot in vitro culture.

## Materials & methods

### Dataset and model development

**Fig. 1:**
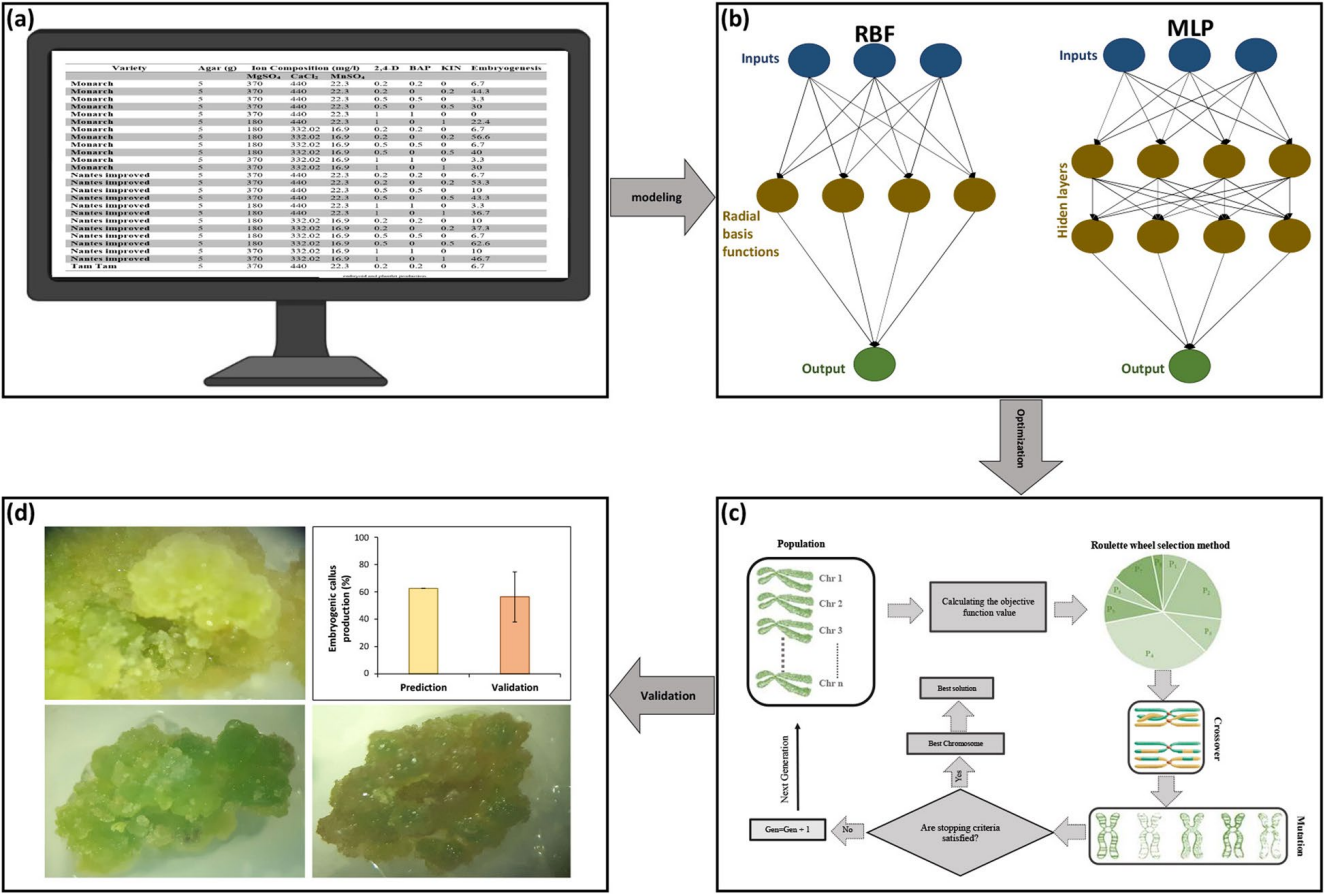
The schematic view of the step-by-step methodology of this study including (**a**) database obtained from previous studies on carrot somatic embryogenesis, (**b**) data modeling using multilayer perceptron (MLP) and radial basis function (RBF), (**c**) optimization process via genetic algorithm (GA), and (**d**) experimental validation test for carrot embryogenic callus production rate

Data for the current investigation was collected from previous carrot somatic embryogenesis studies (Table S1) [29–35]. Carrot cultivars, agar, magnesium sulfate (MgSO_4_), calcium dichloride (CaCl_2_), manganese (II) sulfate (MnSO_4_), 2,4-dichlorophenoxyacetic acid (2,4-D), 6-benzylaminopurine (BAP), and kinetin (KIN) were considered as the input variables, while embryogenic callus production rate (%) was considered as the target variable (Fig. 1a). To detect embryogenesis of calli, calli were examined under microscope and embryogenic calli were distinguished from non-embryogenic calli.

The dataset was employed for modeling, predicting, and optimizing carrot embryogenic callus production. Figure 1 displays the step-by-step procedure of this study. In the current study, MLP and RBF as two well-known ANNs were used to model and predict carrot embryogenic callus production. Before using the ANNs, Box-Cox transformation was employed to normalize the data. To detect outliers, principal component analysis (PCA) was used; however, no outlier was identified. In this study, the five-fold cross-validation approach with 10 repetitions was applied to evaluate the prediction accuracy of the tested ANNs. To evaluate and compare the efficiency and accuracy of the ANNs, *R*^2^ (coefficient of determination), mean bias error (MBE), and root mean square error (RMSE) were employed.

### Multilayer Perceptron (MLP)

MLP is one of the most well-known Artificial Neural Networks. MLP consists of three layers. These three layers include one or more hidden layers, an input layer and an output layer (Fig. 1b). Input and output variables are provided to the network by a supervised training method, which is implemented by MLP. This training set continues until the following equation is minimized:

1 $$E = \frac{1}{K} \sum\limits_{k=1}^{K}\left(y_k-{\hat{y}}_k\right)^2$$

In this formula, the concepts of components are K (the number of data points), yk (the kth observed data), ${\widehat y}_k$) the *k*^*th*^ forecasted data). The three layers in an MLP model with n inputs and m neurons in the hidden layer are determined as follows [37].

2 $$\hat{y} = f\left[ {\sum\limits_{j = 1}^{m} {w_{j} .g(\sum\limits_{i = 1}^{n} {w_{ji} x_{i} + w_{j0} ) + w_{o} } } } \right]$$

Components of the above formula where *x*_*i*_ is the *i*^*th*^ input variable, *w*_*0*_ represents bias related to the neuron of output, *w*_*j0*_ is bias of the *j*^*th*^ neuron of hidden layer, *f* represents transfer functions for the output layer, *g* is the transfer functions for hidden layer, *w*_*ji*_ is the weight connecting the *j*^*th*^ neuron of hidden layer and the *i*^*th*^ input variable, and *w*_*j*_ represents weight linking the neuron of output layer and the *j*^*th*^ neuron of hidden layer.

To determine the main function of MLP, it is necessary to determine its structure. Determining the Hornik et al. (1989) showed that what plays an important role in determining the structure of the MLP is the number of neurons in the hidden layer.number of neurons in each layer and the number of hidden layers is necessary to build this model. In general, by using trial and error, the optimal number of neurons in the hidden layer can be calculated [37]. In the current study, the optimal neuron number in the hidden layer was detected based on trial and error. Also, linear function (purelin) and hyperbolic tangent sigmoid function (tansig) were applied as the transfer functions of output layer and hidden layer, respectively. Moreover, bias and weights were adjusted by using a Levenberg–Marquardt algorithm. The pseudocode of MLP has been described in Table S2.

### Radial Basis Function (RBF)

The basis of radial-based networks that organize statistical Artificial Neural Networks is a three-layer ANN including an input layer, a hidden layer and an output layer, and RBF is one of these networks (Fig. 1b). Statistical neural networks have regression-based approaches (Lin et al. 2020). The Gaussian function in RBF is the most well-known transfer function, which is determined by the following equation:

3 $$f(X_{r} ,X_{b} ) = e^{{ - [\left\| {X_{r} - X_{b} } \right\|*0.8326/h]^{2} }}$$

In this formula, X_r_ (input with unknown output), X_b_ (observed inputs in time b) and h (spread) are. The dependent variable (Yr) is determined by predicting Xr as follows:

4 $$Y_{r} = \sum\limits_{b = 1}^{m} {w_{b} *f(X_{r} ,X_{b} ) + w_{0} }$$

where* w*_*0*_ and ${w}_{j}$ are the bias and weight of linkage between the *b*^th^ hidden layer and the output layer, respectively. The pseudocode of RBF has been described in Table S3 (Jafari and Shahsavar 2020).

### Genetic algorithm optimization

Genetic algorithm (GA) as a single-objective evolutionary optimization algorithm was used for finding the appropriate level of agar, MgSO_4_, CaCl_2_, MnSO_4_, 2,4-D, BAP, KIN, and carrot cultivar to obtain the maximum embryogenic callus production rate. The upper bound and lower bound of the dataset were considered as constraints, and the point with the highest value for embryogenic callus production was recognized as the optimal solution. Roulette wheel as a selection function, 2-point crossover function, and the uniform of mutation function were considered during the optimization process. The crossover rate, generation number, initial population, and mutation rate were respectively set to 0.7, 1000, 200, and 0.04 to obtain the best fitness (Fig. 1c). The pseudocode of GA has been described in Table S4.

### Sensitivity analysis

Sensitivity analysis was conducted to identify the importance degree of agar, MgSO_4_, CaCl_2_, MnSO_4_, 2,4-D, BAP, KIN, and carrot cultivar on the embryogenic callus production rate. In order to evaluate the performance (RMSE) of the developed RBF model, the value of variable sensitivity error (VSE) is measured.

### Validation experiments

To confirm the reliability and accuracy of the developed model, the result obtained from RBF-GA model were tested in the laboratory. Therefore, different parts (leaf, petiole, and stem segments) of in vitro-grown seedling of Nantes improved cultivar were cultured on modified MS (Murashige and Skoog 1962) medium containing 195.23 mg/l MgSO_4_, 330.07 mg/l CaCl_2_, 18.3 mg/l MnSO_4_, 0.46 mg/l 2,4- D, 0.03 mg/l BAP, and 0.88 mg/l KIN. All media had 0.6% agar and 3% sucrose, and the pH of the media was adjusted to 5.8 before autoclaving for 20 min at 120 ℃. All culture boxes were placed in the growth chamber at 25 ± 3 °C under 16-h photoperiod with 50 ± 4 μmol m^−2^ s^−1^ light intensity. The experiment was performed based on the completely randomized design with three replications (each replication contained 5 culture boxes including 5 explants).

## Results

### Modeling and comparative analysis of MLP and RBF

In the current study, MLP and RBF models were used to predict carrot embryogenic callus production based on eight input variables including agar, MgSO_4_, CaCl_2_, MnSO_4_, 2,4-D, BAP, KIN, and carrot cultivar. Data modeling through machine learning algorithms can provide a reliable approach to improve detailed knowledge on carrot embryogenic callus production. The RBF model displayed higher predictive accuracy (R^2^ > 0.67) in both training and testing sets in comparison to MLP (R^2^ > 0.61) for carrot embryogenic callus production (Table 1).

**Table 1:** Performance criteria of artificial neural networks (ANNs) for embryogenic callus production in carrot

Model	Training set				Testing set		
	R^2^	RMSE	MBE		R^2^	RMSE	MBE
RBF	0.833	7.879734	0.311607		0.666	10.23974	3.78125
MLP	0.796	10.324011	0.440372		0613	12.74561	4.35412

*MLP* Multilayer perceptron, *RBF* Radial basis function, *R*^*2*^ Coefficient of determination, *RMSE* Root mean square error, *MBE* Mean bias error

**Fig. 2:**
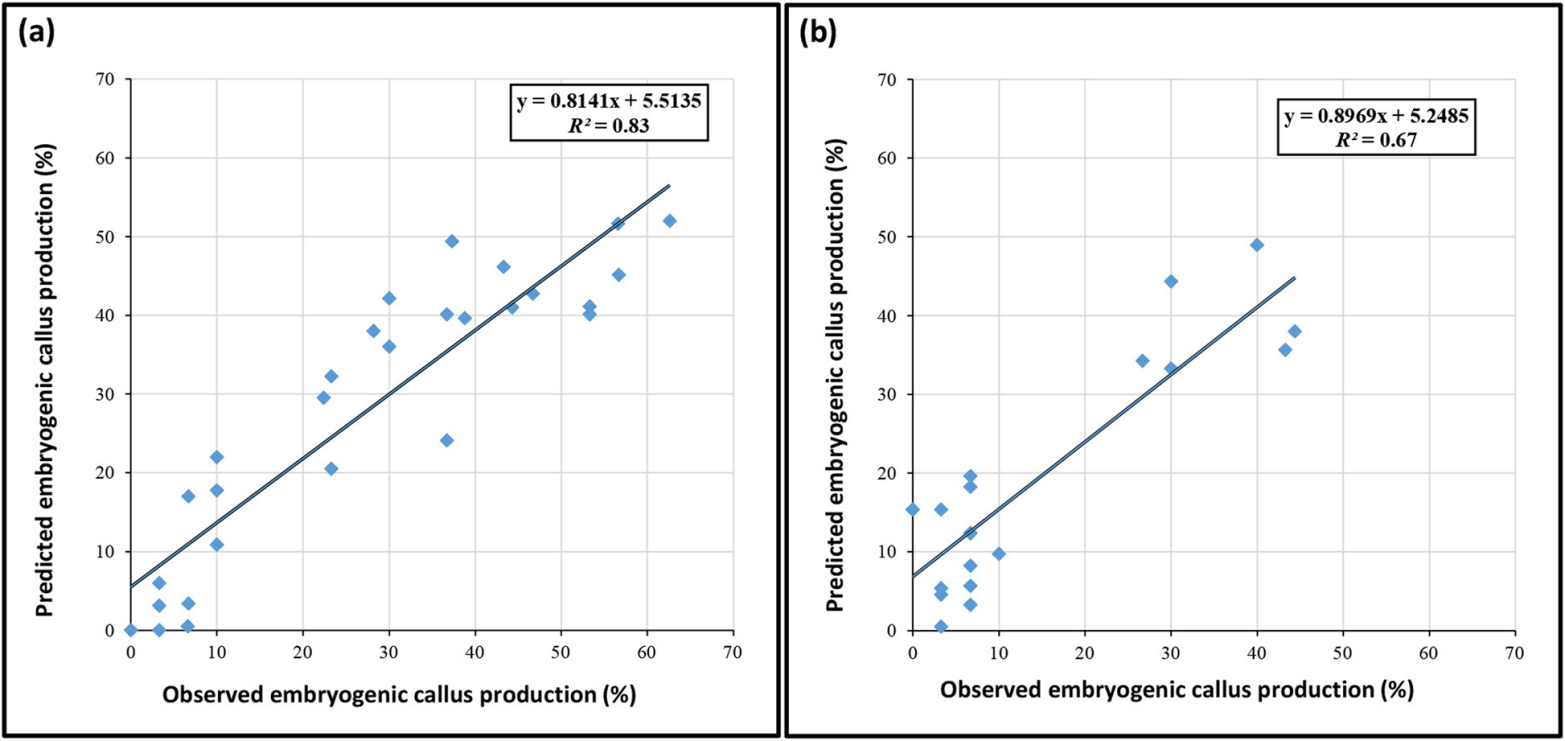
Scatter plot of predicted data against observed data of carrot embryogenic callus production using radial basis function (RBF) in (**a**) training and (**b**) testing sets

Model accuracy was evaluated by RMSE and MBE, which found RBF to be more accurate than MLP (Table 1). Also, the regression lines revealed a good fit correlation between experimental and predicted values for embryogenic callus production rate in the both training (Fig. 2a) and testing (Fig. 2b) sets.

### Sensitivity analysis

The importance of each input in the developed model was assessed via the whole database to evaluate the general variable sensitivity ratio (VSR). The VSR was obtained for the embryogenic callus production rate with respect to agar, MgSO_4_, CaCl_2_, MnSO_4_, 2,4-D, BAP, KIN, and carrot cultivar (Table 2). Sensitivity analysis demonstrated that embryogenic callus production rate was more sensitive to variety, followed by 2,4-D, BAP, KIN, MnSO_4_, CaCl_2_, and MgSO_4_ (Table 2).

**Table 2:** Importance of input variables for embryogenic callus production of carrot according to sensitivity analysis

Item	Inputs							
	Variety	Agar	MgSO_4_	CaCl_2_	MnSO_4_	2,4-D	BAP	KIN
VSR	1.623392	0.999596	1.018606	1.019633	1.020771	1.558631	1.460148	1.310833
Rank	1	8	7	6	5	2	3	4

*VSR* variable sensitivity ratio, *MgSO*_*4*_ magnesium sulfate, *CaCl*_*2*_ calcium dichloride, *MnSO*_*4*_ manganese (II) sulfate, *2,4-D* 2,4-dichlorophenoxyacetic acid, *BAP* 6-benzylaminopurine, *KIN* kinetin

### Optimization via genetic algorithm

Genetic algorithm (GA) as an evolutionary single-objective optimization algorithm was linked to the RBF (the most accurate model in this study) in order to find the optimal level of agar, MgSO_4_, CaCl_2_, MnSO_4_, 2,4-D, BAP, KIN, and carrot cultivar for obtaining the maximum embryogenic callus production rate. According to the RBF-GA result (Table 3), the highest somatic embryogenesis rate (62.5%) can be obtained from Nantes improved cultivar cultured on medium containing 195.23 mg/l MgSO_4_, 330.07 mg/l CaCl_2_, 18.3 mg/l MnSO_4_, 0.46 mg/l 2,4- D, 0.03 mg/l BAP, and 0.88 mg/l KIN.

**Table 3:** The optimal level of input variables for maximizing embryogenic callus production in carrot through genetic algorithm (GA)

Input variables								Fitness function
Variety	Agar (g)	MgSO_4_ (mg/l)	CaCl_2_ (mg/l)	MnSO_4_ (mg/l)	2,4-D (mg/l)	BAP (mg/l)	KIN (mg/l)	Embryogenic callus production (%)
Nantes improved	6.406556	195.23	330.07	18.3	0.46	0.032262	0.875449	62.5

*MgSO*_*4*_ magnesium sulfate, *CaCl*_*2*_ calcium dichloride, *MnSO*_*4*_ manganese (II) sulfate, *2,4-D* 2,4-dichlorophenoxyacetic acid, *BAP* 6-benzylaminopurine, *KIN* kinetin

### Validation experiments

The results of the validation experiment demonstrated that there was no significant difference between the predicted optimized result and experimental data (Fig. 1d). It is notable that the highest embryogenic callus production was obtained from leaf segments.

## Discussion

The somatic embryogenesis in carrot has been previously studied [21, 29, 30, 32–35]. Establishing carrot somatic embryogenesis was associated with different obstacles such as low efficiency of somatic embryogenesis, chimeric callogenesis consisting of both non-embryogenic and embryogenic calli [21, 29, 30, 32–35]. Data mining approaches may help in reducing trial and errors in the process of optimizing carrot somatic embryogenesis. A hybrid of ANN-GA can be considered as promising method to model and optimize somatic embryogenesis protocols [27]. Although there are no reports to use ANN-GA in in vitro culture of carrot, several studies have previously proved the reliability and accuracy of ANN-GA to predict and optimize different in vitro culture processes such as in vitro sterilization [12, 13], callogenesis [36], somatic embryogenesis [18], shoot regeneration [19], and secondary metabolite production [23] in other plants.

Although most plant tissue culture studies have employed MLP for modeling and predicting in vitro culture systems, according to our results, RBF had better performance than MLP for modeling and predicting carrot embryogenic callus production. Several studies have demonstrated that RBF had better accuracy than other MLP. In line with our results, Hesami, et al. [14] showed that RBF had better efficiency than MLP for modeling in vitro seed germination of cannabis. Also, Jafari and Shahsavar [37] compared the efficiency of RBF and MLP for modeling and predicting morphological responses of lime under water deficiency and reported that RBF had better predictive performance than MLP.

The results of the validation experiment showed that there was no significant difference between experimental results and predicted optimized results for embryogenic callus production of carrot. Based on our results, the highest somatic embryogenesis rate was obtained from Nantes improved cultivar cultured on medium containing 195.23 mg/l MgSO_4_, 330.07 mg/l CaCl_2_, 18.3 mg/l MnSO_4_, 0.46 mg/l 2,4- D, 0.03 mg/l BAP, and 0.88 mg/l KIN. In line with our result, previous studies showed that type of cultivar, type and concentration of PGRs, and concentration of magnesium, calcium, and manganese play a pivotal role in the embryogenic callus production of carrot [29–35]. For instance, Rabiei, et al. [33] reported that reducing MgSO_4_, CaCl_2_, and MnSO_4_ in MS media to 180 mg/l, 332.02 mg/l, and 16.9 mg/l, respectively, resulted in a higher embryogenic callus production rate in carrot in comparison with MS medium with 370 mg/l MgSO_4_, 400 mg/l CaCl_2_, and 22.3 mg/l MnSO_4_.

It is well documented that the level of PGRs, in particular auxin and cytokinin, play an important role in embryogenic callus production [38]. 2,4-D as one of the most important synthetic auxins plays an important role in tissue culture and embryogenic cell systems, and callus induction in plant tissue culture studies [39]. Also, 2.4-D has a positive impact on the molecular and physiological process of callus by inducing specific proteins, regulating the endogenous IAA metabolism, and controlling DNA methylation [38]. The balance between auxin and cytokinin is another main factor in embryogenic callus production. Zhao [40–44] reported that the ratio between auxins and cytokinins plays an important role in in vitro developmental processes.

## Conclusions

Carrot is a model plant in genetic engineering works and recombinant drugs, and therefore it is an important plant in research works. Also, in this research, for the first time, embryogenesis in carrot (Daucus carota L.) using Genetic algorithm (GA) and data mining technology has been reviewed and analyzed.

Embryogenic callus production as a multifactorial process is affected by several factors such as genotypes, explants, PGRs, and medium composition, which limits the ability to obtain reliable and replicable protocols. Optimizing in vitro conditions can be considered as one of the main steps to establish a high-frequency regeneration protocol. Recently, a hybrid of machine learning and optimization algorithm has been successfully applied for modeling and optimizing different in vitro culture systems. In this study, two types of ANN (MLP and RBF) in combination with GA was implemented to model and forecast embryogenic callus production in carrot. Our results demonstrated that the RBF-GA model can accurately model and optimize carrot embryogenic callus production. Generally, the results showed that data mining through RBF-GA can be considered as a robust approach for modeling and optimizing embryogenic callus production without lots of bench work experiments. Further experimental work is required to validate the reliability and accuracy of this approach on other in vitro culture systems (e.g., shoot proliferation, indirect organogenesis, androgenesis).

Sensitivity analysis demonstrated that embryogenic callus production rate was more sensitive to variety, followed by 2,4-D, BAP, KIN, MnSO_4_, CaCl_2_, and MgSO_4._

One of the success factors of tissue culture is the use of the used variety. Sundararajan et al., used *D.carota* L., cv. Kurado [45], Tavares et al., used *D. carota* subsp. *Halophilus* [46] and Kamada et al., used *Daucus carota* L. cv. US-Harumakigosun [35]. However, used variety in this research was Nantes.

2,4-D is a type of auxin necessary for callus induction. Previous study shows that high concentration of 2,4-D can block normal callus induction [47] and cause disrupt natural genetic and physiological processes [48]. Hardegger et al., in 1998 used of 0.1 mg/l 2,4-D for callus induction in carrot [49]. Marquet-Blouin et al*.,* 2003 and Yau et al*.,* in 2012 indicate 1 mg/l 2,4-D most effective in callus induction in carrot [50]. In another study by Rabiei et al*.,* in 2010 shown high callus induction obtain in 0.2 mg/l 2,4-D [46].

6-Benzylaminopurine (BAP) or benzyl adenine (BA) is a first-generation synthetic cytokinin. BAP has a different role in tissue culture of plant, including elicits plant growth and development responses, setting blossoms and stimulating fruit richness by stimulating cell division. Pant et al*.,* Rabiei et al*.,* in 2010 indicate 1 mg/l BAP needed for callus induction [46]. Heidegger et al*.,* in 1998 used 0.5 mg/l BAP for callus induction. This result similar whit result in this study [49].

Kinetin is a cytokinin-like synthetic compound that regulates cell growth in plants. Kinetin was the first cytokinin discovered. It is also adenine-based. Kinetin is often used in plant tissue culture for inducing formation of callus (in conjunction with auxin) and to regenerate shoot tissues from callus (with lower auxin concentration [51].

MgSO_4_, CaCl_2,_ and MnSO_4_ are macroelements nutrient. These elements are essential in the growth of explants in tissue culture [51].

Magnesium is an essential component of the chlorophyll molecule. This compound is vital for the activity of several nonspecifically enzymes and role in the transfer of phosphates. MgSO_4_ the chlorophyll molecule as a the central atom in the porphyrin structure. A magnesium ion in plants is mobile and roles as a cation, balancing and neutralizing anions and organic acids. Often, MgSO_4_ is used as a unique source of both magnesium and sulfate ions [52].

Calcium roles as a cofactor with different enzymes and for cell wall synthesis is essential. Calcium deficiency cause shoots tip necrosis. Used common form of calcium in plant tissue culture is calcium chloride. Calcium roles of pH cellular and a regulator in the source, sink translocation of Carbohydrates too [53].

Manganese (Mn) is a cofactor. It is needed for some enzyme reactions, particularly in respiration and photosynthesis reaction. Usually added form in plant medium is manganese sulfate [52].

## Supplementary Information

Supplementary Material 1.

## Data Availability

All data generated or analysed during this study are included in this published article [and its supplementary information files].

## Data availability

Data is provided within the manuscript or supplementary information files.
