# Supplementary material for: Choosing an appropriate somatic embryogenesis medium of carrot (Daucus carota L.) by data mining technology
Source: BMC Biotechnol. 2024 Sep 27;24:68. doi: 10.1186/s12896-024-00898-7 (PMC11428924; doi:10.1186/s12896-024-00898-7)
Supplement: Supplementary file 1 — Supplementary Material 1. [file 12896_2024_898_MOESM1_ESM.docx]

**Choosing an appropriate somatic embryogenesis medium of carrot (*Daucus carota* L.) by data mining technology**

Masoumeh Fallah Ziarani^1^, Masoud Tohidfar^1*^, Mohsen Hesami^2^

Masoumeh Fallah Ziarani^1^, Masoud Tohidfar^1*^, Mohsen Hesami^2^

^1^ Department of Cell & Molecular Biology, Faculty of Life Sciences & Biotechnology, Shahid Beheshti University, Tehran, 19839-69411, Iran

^2^ Department of Plant Agriculture, University of Guelph, Guelph, ON, Canada

* Corresponding Author:

Masoud Tohidfar

Department of Cell & Molecular Biology, Faculty of Life Sciences & Biotechnology, Shahid Beheshti University, Tehran, 19839-69411, Iran

Email address: m_tohidfar@sbu.ac.ir

| **Table S1**. Database obtained from previous studies on carrot somatic embryogenesis | | | | | | | | |
| --- | --- | --- | --- | --- | --- | --- | --- | --- |
| Inputs | | | | | | | | Output |
| Variety | Agar (g) | MgSO_4_ (mg/l) | CaCl_2_ (mg/l) | MnSO_4_ (mg/l) | 2,4-D (mg/l) | BAP (mg/l) | KIN (mg/l) | Embryogenic callus production (%) |
| Monarch | 5 | 370 | 440 | 22.3 | 0.2 | 0.2 | 0 | 6.7 |
| Monarch | 5 | 370 | 440 | 22.3 | 0.2 | 0 | 0.2 | 44.3 |
| Monarch | 5 | 370 | 440 | 22.3 | 0.5 | 0.5 | 0 | 3.3 |
| Monarch | 5 | 370 | 440 | 22.3 | 0.5 | 0 | 0.5 | 30 |
| Monarch | 5 | 370 | 440 | 22.3 | 1 | 1 | 0 | 0 |
| Monarch | 5 | 180 | 440 | 22.3 | 1 | 0 | 1 | 22.4 |
| Monarch | 5 | 180 | 332.02 | 16.9 | 0.2 | 0.2 | 0 | 6.7 |
| Monarch | 5 | 180 | 332.02 | 16.9 | 0.2 | 0 | 0.2 | 56.6 |
| Monarch | 5 | 180 | 332.02 | 16.9 | 0.5 | 0.5 | 0 | 6.7 |
| Monarch | 5 | 180 | 332.02 | 16.9 | 0.5 | 0 | 0.5 | 40 |
| Monarch | 5 | 370 | 332.02 | 16.9 | 1 | 1 | 0 | 3.3 |
| Monarch | 5 | 370 | 332.02 | 16.9 | 1 | 0 | 1 | 30 |
| Nantes improved | 5 | 370 | 440 | 22.3 | 0.2 | 0.2 | 0 | 6.7 |
| Nantes improved | 5 | 370 | 440 | 22.3 | 0.2 | 0 | 0.2 | 53.3 |
| Nantes improved | 5 | 370 | 440 | 22.3 | 0.5 | 0.5 | 0 | 10 |
| Nantes improved | 5 | 370 | 440 | 22.3 | 0.5 | 0 | 0.5 | 43.3 |
| Nantes improved | 5 | 180 | 440 | 22.3 | 1 | 1 | 0 | 3.3 |
| Nantes improved | 5 | 180 | 440 | 22.3 | 1 | 0 | 1 | 36.7 |
| Nantes improved | 5 | 180 | 332.02 | 16.9 | 0.2 | 0.2 | 0 | 10 |
| Nantes improved | 5 | 180 | 332.02 | 16.9 | 0.2 | 0 | 0.2 | 37.3 |
| Nantes improved | 5 | 180 | 332.02 | 16.9 | 0.5 | 0.5 | 0 | 6.7 |
| Nantes improved | 5 | 180 | 332.02 | 16.9 | 0.5 | 0 | 0.5 | 62.6 |
| Nantes improved | 5 | 370 | 332.02 | 16.9 | 1 | 1 | 0 | 10 |
| Nantes improved | 5 | 370 | 332.02 | 16.9 | 1 | 0 | 1 | 46.7 |
| Tam Tam | 5 | 370 | 440 | 22.3 | 0.2 | 0.2 | 0 | 6.7 |
| Tam Tam | 5 | 370 | 440 | 22.3 | 0.2 | 0 | 0.2 | 38.8 |
| Tam Tam | 5 | 370 | 440 | 22.3 | 0.5 | 0.5 | 0 | 0 |
| Tam Tam | 5 | 370 | 440 | 22.3 | 0.5 | 0 | 0.5 | 30 |
| Tam Tam | 5 | 180 | 440 | 22.3 | 1 | 1 | 0 | 0 |
| Tam Tam | 5 | 180 | 440 | 22.3 | 1 | 0 | 1 | 26.7 |
| Tam Tam | 5 | 180 | 332.02 | 16.9 | 0.2 | 0.2 | 0 | 6.7 |
| Tam Tam | 5 | 180 | 332.02 | 16.9 | 0.2 | 0 | 0.2 | 56.7 |
| Tam Tam | 5 | 180 | 332.02 | 16.9 | 0.5 | 0.5 | 0 | 6.7 |
| Tam Tam | 5 | 180 | 332.02 | 16.9 | 0.5 | 0 | 0.5 | 43.3 |
| Tam Tam | 5 | 180 | 332.02 | 16.9 | 1 | 0 | 0 | 3.3 |
| Tam Tam | 5 | 180 | 332.02 | 16.9 | 1 | 0 | 1 | 23.3 |
| Vilmorn | 5 | 180 | 332.02 | 16.9 | 0.2 | 0.2 | 0 | 6.7 |
| Vilmorn | 5 | 180 | 332.02 | 16.9 | 0.2 | 0 | 0.2 | 36.7 |
| Vilmorn | 5 | 180 | 332.02 | 16.9 | 0.5 | 0.5 | 0 | 3.3 |
| Vilmorn | 5 | 180 | 332.02 | 16.9 | 0.5 | 0 | 0.5 | 28.2 |
| Vilmorn | 5 | 180 | 332.02 | 16.9 | 1 | 0 | 0 | 3.3 |
| Vilmorn | 5 | 180 | 332.02 | 16.9 | 1 | 0 | 1 | 23.3 |
| Vilmorn | 5 | 180 | 332.02 | 16.9 | 0.2 | 0.2 | 0 | 10 |
| Vilmorn | 5 | 180 | 332.02 | 16.9 | 0.2 | 0 | 0.2 | 53.3 |
| Vilmorn | 5 | 180 | 332.02 | 16.9 | 0.5 | 0.5 | 0 | 6.6 |
| Vilmorn | 5 | 180 | 332.02 | 16.9 | 0.5 | 0 | 0.5 | 44.4 |
| Vilmorn | 5 | 180 | 332.02 | 16.9 | 1 | 1 | 0 | 3.3 |
| Vilmorn | 5 | 180 | 332.02 | 16.9 | 1 | 0 | 1 | 30 |
| us-Harumakigosum | 5 | 180 | 332.02 | 16.9 | 0 | 0 | 0 | 0 |
| us-Harumakigosum | 5 | 180 | 332.02 | 16.9 | 0 | 0 | 0 | 0 |
| us-Harumakigosum | 5 | 180 | 332.02 | 16.9 | 0 | 0 | 0 | 0 |
| us-Harumakigosum | 5 | 180 | 332.02 | 16.9 | 0 | 0 | 0 | 0 |
| us-Harumakigosum | 5 | 180 | 332.02 | 16.9 | 0.001 | 0 | 0 | 0 |
| us-Harumakigosum | 5 | 180 | 332.02 | 16.9 | 0.01 | 0 | 0 | 14 |
| us-Harumakigosum | 5 | 180 | 332.02 | 16.9 | 0.1 | 0 | 0 | 14 |
| us-Harumakigosum | 5 | 180 | 332.02 | 16.9 | 1 | 0 | 0 | 0 |
| us-Harumakigosum | 5 | 180 | 332.02 | 16.9 | 10 | 0 | 0 | 0 |
| us-Harumakigosum | 5 | 180 | 332.02 | 16.9 | 0 | 0 | 0 | 0 |
| us-Harumakigosum | 5 | 180 | 332.02 | 16.9 | 0 | 0 | 0 | 0 |
| us-Haru+A85makigosum | 5 | 180 | 332.02 | 16.9 | 0 | 0 | 0 | 0 |
| us-Haru+A85makigosum | 8 | 180 | 332.02 | 16.9 | 0 | 0 | 0 | 0 |
| us-Haru+A85makigosum | 8 | 180 | 332.02 | 16.9 | 0 | 0 | 0 | 0 |
| us-Haru+A85makigosum | 8 | 180 | 332.02 | 16.9 | 0 | 0 | 0 | 0 |
| us-Haru+A85makigosum | 8 | 180 | 332.02 | 16.9 | 0 | 0 | 0 | 0 |
| us-Haru+A85makigosum | 8 | 180 | 332.02 | 16.9 | 0.001 | 0 | 0 | 0 |
| us-Haru+A85makigosum | 8 | 180 | 332.02 | 16.9 | 0.01 | 0 | 0 | 14 |
| us-Haru+A85makigosum | 8 | 180 | 332.02 | 16.9 | 0.1 | 0 | 0 | 14 |
| us-Haru+A85makigosum | 8 | 180 | 332.02 | 16.9 | 1 | 0 | 0 | 0 |
| us-Haru+A85makigosum | 8 | 180 | 332.02 | 16.9 | 10 | 0 | 0 | 0 |
| us-Haru+A85makigosum | 8 | 180 | 332.02 | 16.9 | 0 | 0.1 | 0 | 0 |
| us-Haru+A85makigosum | 8 | 180 | 332.02 | 16.9 | 0 | 1 | 0 | 0 |
| MgSO_4_: magnesium sulfate, CaCl_2_: calcium dichloride, MnSO_4_: manganese (II) sulfate, 2,4-D: 2,4-dichlorophenoxyacetic acid, BAP: 6-benzylaminopurine, KIN: kinetin | | | | | | | | |

| **Table S2**. The pseudocode of Multilayer Perceptron (MLP) |
| --- |
| Choosing an initial weight vector *~w*  initialize minimization approach  **while** error did not converge **do**  **for all** (*~x, ~d*) *ϵ D* **do**  apply *~x* to network and calculate the network output  calculate *ꝺe* (*~x*)  **end for**  calculate *ꝺE* (*~D*)  for all weights summing over all training patterns  perform one update step of the minimization approach  **end while** |

| **Table S3**. The pseudocode of Radial basis function (RBF) |
| --- |
| **Input:**  sequence of labeled training patterns *Z*= [(*x_1_, y_1_*), …, (*x_l_, y_l_*)]  number of RBF centers *K*  regularization constant *ƛ*  Number of iterations *O*  **Initialize:**  run *K*-means clustering to find initial values for *µ_k_* and determine *σ_k_*, *k*=1, 2, …, K, as the distance between *µ_k_* and closest *µ_i_* (i ≠ k).  **Do for *o*= 1 : *O*,**  compute optimal output weights *w* = (*G^T^G + 2* $\frac{ƛ}{l}$ *I*)^-1^ *G^T^y*  compute gradients $\frac{\delta}{\delta\mu k}E$ and $\frac{\delta}{\delta\mu k}E$ with optimal *w* and form a gradient vector *v*  estimate the conjugate direction $\bar{v}$ with Gaussian function,  perform a line search to find the minimizing step size *ꝺ* in direction $\bar{v};$ in each evaluation of *E* recompute the optimal weights *w* as in line 1  update *µ_k_* and *σ_k_* with $\bar{v}$ and *ꝺ*  **while end: Output** |

| **Table S4**. The pseudocode of Genetic Algorithm (GA) |
| --- |
| Given:  -*nP*: base population size.  -*nI*: number of iterations.  -*rC*: rate of crossover.  -*rM*: rate of mutations.  generate initial population of size *nP*.  evaluate initial population according to the fitness function.  **While** (*current_iteration ≤ nI*)  // Breed *rC × nP* new solutions.  Select two parent solutions from current population.  From offspring’s solution via crossover.  **If** (*rand*(0.0, 1.0) < *rM*)  mutate the offspring’s solutions.  **end if**  evaluate each child solution according to the fitness function.  add offspring’s to population.  // population size is now *MaxPop*= *nP* × (1 + *rC*)  remove the *rC* × *nP* least-fit solutions from population.  **end while**  output the global best solution |
